# Supplementary material for: Content-rich biological network constructed by mining PubMed abstracts
Source: BMC Bioinformatics. 2004 Oct 8;5:147. doi: 10.1186/1471-2105-5-147 (PMC528731; doi:10.1186/1471-2105-5-147)
Supplement: Additional File 2 — The original results of the above study (non-essential files are deleted to keep the file size under the limit set by BMC bioinformatics). [file 1471-2105-5-147-S2.bz2 › chilibotAdditionalFile2/dip05/255ID7911228E1014/html/CDKN1A_PCNA.html]

 


 **CDKN1A** and **PCNA** 
  
Found 1649 abstracts in PubMed, retrieved 05.  
 

 What does Google say? 
 PDF only 
| .edu only 

---

**Interactive relationship** (e.g. stimulation, inhibition, etc)

**Inhibitory relationship**- p21 Waf1  [ **CDKN1A** ]  Cip1  [ **CDKN1A** ]  exerts a key role in driving this G 2 exit both by inhibiting cyclin  [ **PCNA** ]  B1 Cdk1 and cyclin  [ **PCNA** ]  A Cdk1 2 complexes, which control G 2 M progression, and by blocking the phosphorylation of pRb family proteins.  Ref: 12881433 EMBO JEMBO J,
**Neutral relationship**

**Non-interactive relationship** (e.g. studied together, co-existance, homology, etc.)

- AIMS To correlate the expression of a series of apoptotic and oncogene markers including p53, Bcl 2, BAX, Bcl XL, p21WAF, 1 CIP1  [ **CDKN1A** ] , cyclin  [ **PCNA** ]  D1, HER 2 neu in thymic epithelial tumours with histological type, stage and resectability and to determine whether the information on HER 2 neu would be valuable in identifying patients who are eligible for anti HER 2 neu treatment.  Ref: 12877732 Histopathology, 2003
- Both 5% human fecal water and 4 micro mol L selenite greatly increased the mRNA level of the cyclin  [ **PCNA** ]  dependent kinase inhibitor gene p21 waf1  [ **CDKN1A** ] .  Ref: 12888658 J Nutr, 2003
- Most thymomas and thymic carcinomas were negative for Bcl XL, p21WAF, 1 CIP1  [ **CDKN1A** ]  and cyclin  [ **PCNA** ]  D1.  Ref: 12877732 Histopathology, 2003
- These events were accompanied by down regulation of the Raf 1 MEK ERK pathway as well as diminished expression of Bcr Abl and cyclin  [ **PCNA** ]  D1, cleavage of p21 CIP1  [ **CDKN1A** ]  and pRb, and induction of the stress related kinases JNK and p38 MAPK.  Ref: 12893773 BloodBlood, 2003
- These deletions define a genomic region that includes cki 1 and cki 2, adjacent genes encoding members of the Cip  [ **CDKN1A** ]  Kip family of cyclin  [ **PCNA** ]  dependent kinase inhibitors.  Ref: 12885569 Dev Biol, 2003
